# Knowledge-based radiation therapy treatment planning decision support system for head and neck cancer utilizing multi-organ constellation matching

**DOI:** 10.1007/s11548-025-03554-3

**Published:** 2025-11-27

**Authors:** Trent Benedick, Stephanie Zhou, Jorge Solis Galvan, John Asbach, Ryan H. B. Smith, Anh H. Le, Brent Liu

**Affiliations:** 1 Image Processing and Informatics Laboratory, Department of Biomedical Engineering, University of Southern California, 1042 Downey Way, Los Angeles, CA 90089 USA; 2 Roswell Park Comprehensive Cancer Center, Elm and Carlton Streets, Buffalo, NY 14203 USA; 3 Jacobs School of Medicine and Biomedical Sciences, State University of New York at Buffalo, 955 Main Street, Buffalo, NY 14203 USA; 4 Department of Radiation Oncology, University of Minnesota, 516 Delaware St SE, Minneapolis, MN 55455 USA; 5 Department of Radiation Oncology, Cedars-Sinai Medical Center, 8700 Beverly Blvd, Los Angeles, CA 90048 USA

**Keywords:** Radiation therapy, Treatment planning support, Decision support, Knowledge-based treatment planning, Web-based application, Imaging informatics, Informatics infrastructure

## Abstract

**Purpose:**

Radiotherapy treats cancers through precise delivery of radiation to target volumes. Radiotherapy treatment plans, prescribing the delivery of therapeutic radiation, are presently created primarily from clinical experience and application of clinical protocols through trial-and-error rather than standardized quantitative methods. We developed an informatics infrastructure and decision support system to assist during treatment plan creation by providing access to applicable retrospective radiotherapy cases.

**Methods:**

Radiotherapy treatment planning is based in part on tumor position and spatial relationships to surrounding structural anatomy. Our system data mines retrospective cases to identify cases and treatment plans with similar tumor position and surrounding anatomy (i.e., multi-organ-tumor constellation geometry) for clinicians to use as references during treatment plan creation. The system is based on a database of 390 DICOM RT dosimetry digital radiotherapy datasets with associated extracted quantitative features. Using data mining techniques, overall similarity between cases is calculated with features extracted from tumor volumes and organs at risk (OAR).

**Results:**

We implemented our radiotherapy treatment planning decision support system with a full-stack infrastructure, including a database of best practice retrospective cases, an algorithmic backend for feature extraction and similarity calculation, and a frontend web application for clinical use. Clinicians can upload current planning cases to the web application whereupon their similarity to knowledge base cases is calculated, and the most similar are presented to clinicians for selection as references during current treatment plan creation.

**Conclusions:**

This radiotherapy treatment planning decision support system, by providing access to geometrically similar retrospective best practice reference cases, presents a novel tool to improve treatment planning. Development of a full system infrastructure increases standardization, facilitates creation of high quality plans, and assists clinicians, particularly during the critical initial beam configuration stage.

## Introduction

Radiotherapy treats cancers by destroying malignant tissues, however, this process can also kill healthy tissues, causing deleterious effects. Therefore, the challenge of radiotherapy is to deliver a dose sufficient for tumor destruction while simultaneously minimizing exposure to surrounding organs at risk (OARs) sufficiently to prevent adverse outcomes.

To address this challenge, modern radiotherapy systems have been developed that are capable of delivering highly conformal, precise, and accurate treatments [1, 2]. These technological advances have frequently left treatment planning as the largest hurdle to quality plan delivery. Therefore, many treatment planning support tools have been developed to facilitate various steps of the complex treatment planning workflow [1, 3]. In this paper, we present a treatment planning decision support infrastructure designed as a platform for the development, testing, and implementation of planning algorithms for identifying similar radiotherapy cases for use as references during plan creation.

### Clinical background

The increasing complexity of computer controlled radiation delivery machines, which offer numerous parameters for control, necessitate the development of medical imaging informatics infrastructures (MIII). The utilization of MIII for planning and delivery has transformed radiotherapy into a software defined system. Modern treatment planning support tools improve plan creation by using the available data rich environment, including 3D image-based dose simulations and the enhanced delivery accuracy availed by image-guided radiotherapy (IGRT), advanced multileaf collimators (MLCs), and gantry control. The use of 3D planning images for anatomical, dose simulations, and the options afforded by the MIII and the software defined nature of radiotherapy delivery have been key enablers for the development of new treatment modalities and planning support tools [1–6].

**Fig. 1:**
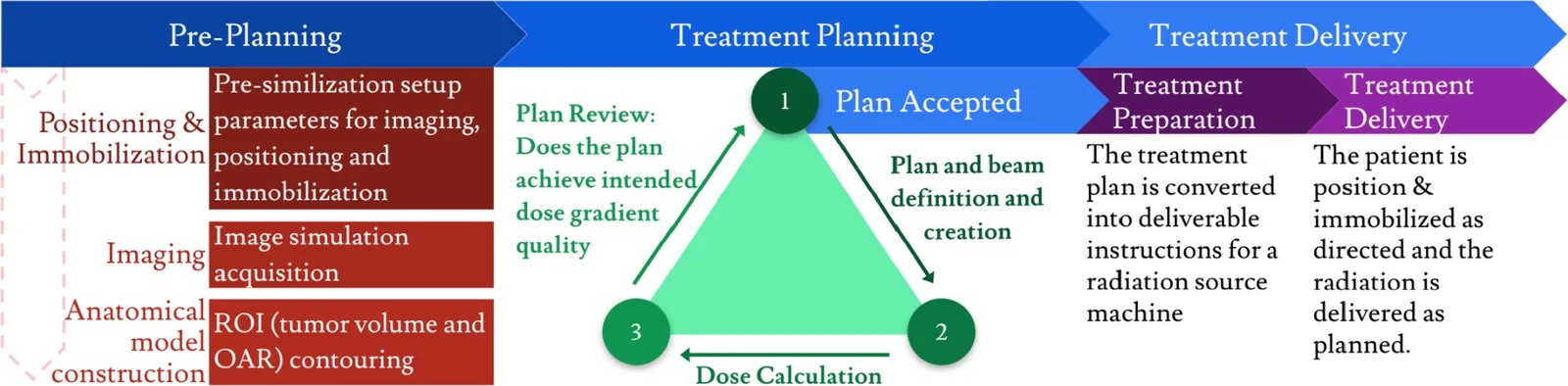
General clinical radiotherapy treatment workflow

Current treatment planning support tools have become essential, fully integrated components of the standard clinical process, facilitating plan creation at various steps of the clinical workflow (Fig. 1). These tools support critical functions such as auto-segmentation, automated dose optimization, and knowledge-based treatment planning (KBP). Work in automated ROI segmentation helps to control ROI volumes from 3D medical images, partially automating a task that is otherwise a manual part of the clinical workflow. Another popular area of development is automated dose optimization and dose-volume histogram (DVH) prediction, which is a key enabling element of intensity-modulated radiotherapy (IMRT). These advances, including intensity-modulated radiotherapy, were made possible by the 3D dose simulations and other informatics capabilities of the MIII and software defined radiotherapy machines [1, 4–9].

Knowledge-based treatment planning is another area of research that utilizes actionable insights from retrospective case datasets to support and optimize treatment planning. Most KBP methods rely on dose simulation comparisons with retrospective cases to predict more optimal dose gradients. Successful commercial implementations exist, such as Varian’s Rapidplan (Varian Medical Systems, Palo Alto, CA, USA), which uses features like ROI registration, target size, overlap area, and dose averaging to automate DVH prediction for plan optimization [10–12]. A review of literature from the last 15 years identifies over 165 published papers on KBP, with many successful approaches focusing on DVH prediction based on dose comparisons or averages [7, 8]. Some methods have partially included anatomical features, such as OAR overlaps, shape, size, and direct registration of ROIS, as part of their DVH prediction models [13–15]. A few studies have taken this a step further by using relational measures to describe the spatial relationship between an OAR and a target volume. However, most of these relational methods have largely been limited to considering single target-OAR pairs and do not construct a holistic model of the GTV-OAR constellation geometry [16–20]. Therefore, we present a KBP infrastructure to enable, test, and implement models which will match cases based on the spatial distributional geometric relationship of the target volumes to all surrounding OARs, what we call the GTV-OAR constellation.

### Proposed decision support infrastructure

**Fig. 2:**
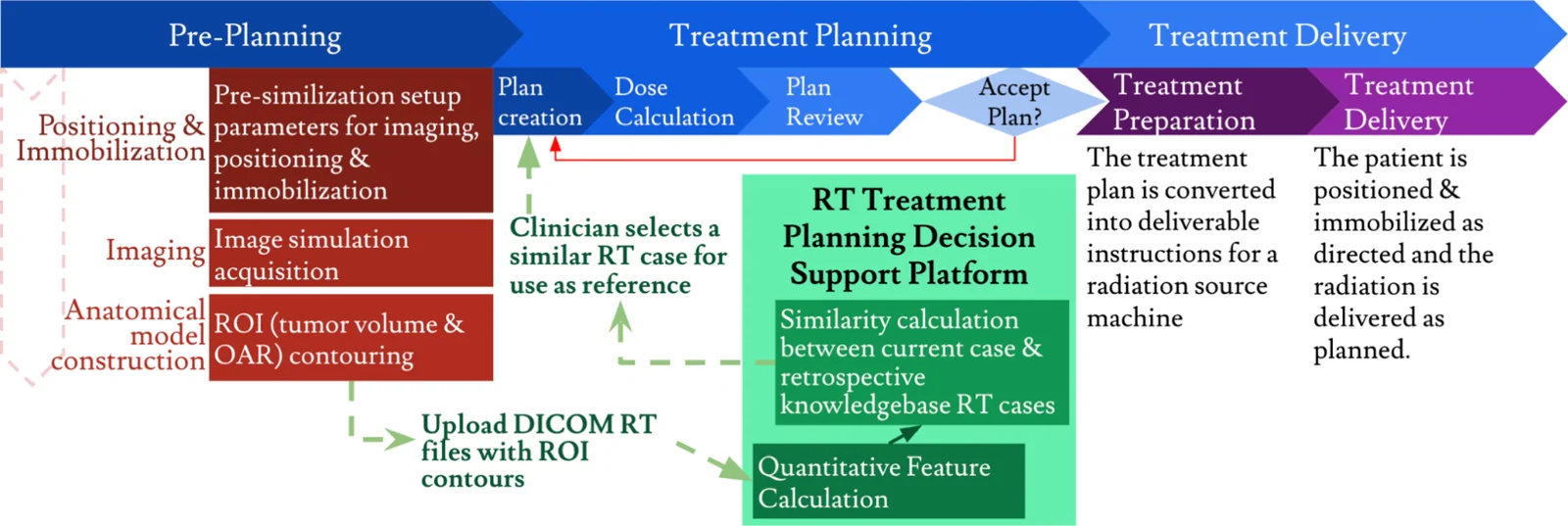
System workflow intervention to clinical radiotherapy treatment workflow. Workflow intervention is in green

The purpose of this work was to develop a novel treatment planning support infrastructure to enable development, testing, and implementation of KBP support tools based on holistic geometric anatomical models. This system matches current planning cases to retrospective best practice cases based on similarity of their GTV-OAR constellation geometry and provides the treatment plans of the similar retrospective cases to serve as references and templates (Fig. 2). This infrastructure follows the trends of previous work by utilizing existing MIII and 3D anatomy simulations to support modeling and comparison of GTV-OAR constellation geometry. This platform is designed to enable exploration, development, and clinical implementation of similarity matching algorithms using holistic geometry models.

The KBP models this system implements allows for further development and exploration of modeling anatomical geometric relationships between target volumes and OARs. The key enabling element of our infrastructure is its ability to calculate quantitative features between the GTV and all OARs, and to store and structure these features relationally from multi-institutional knowledgebase datasets for knowledge-based planning. By calculating quantitative features from the GTV to every OAR, our infrastructure can construct and store a holistic relational representation of the GTV-OAR constellation geometry that is equivalently comparable between cases, even with anatomical variation, a capability that direct surface registration models lack. The data pipeline matches corresponding quantitative features from each GTV-OAR pair between cases, and the overall similarity is calculated from these comparisons. The overall similarity is weighted and normalized based on the number of OARs in common between the two cases, to enable equivalent comparison of calculated similarity across cases. Because it has been demonstrated that OARs with similar spatial relationships to target volumes have similar DVHs, identifying cases by GTV-OAR constellation geometry is a valuable approach [17, 18]. While the KBP model this infrastructure implements considers all GTV-OAR relationships evenly, by retrieving cases with similar constellation geometries, the system identifies clinician-created plans with appropriate differential consideration to each OAR based on factors like serial or parallel criticality or inhomogeneities in tissue between targets and OARs [17, 18].

Our KBP infrastructure retrieves retrospective best practice cases by using GTV-OAR constellation similarity matching, the plans for which can then be used as references during beam number selection and angle or arc conception. The method provides support during a step that is still largely a manual, trial-and-error process, especially in complex instances such as some non-coplanar or SBRT cases. Tools in development for beam configuration support are often complicated, computationally costly and sometimes still don’t assist in the initial conception of beam configurations [21–24]. While planning for many pathologies and treatment modalities is fairly standardized and automated, instances and modalities remain where delineation of beam field angles and arcs is a persisting challenge and art. In these cases, such as some non-coplanar SBRT or proton therapies, reference to template configurations used for similarly positioned target volumes is particularly valuable. Because cases with similar target-OAR anatomical relationships tend to receive similar doses, identifying cases with similar target-OAR constellations will present treatment and dose plans that reflect the clinical importance and implications of different GTV-OAR relationships.

## Methods

### System architecture

**Fig. 3:**
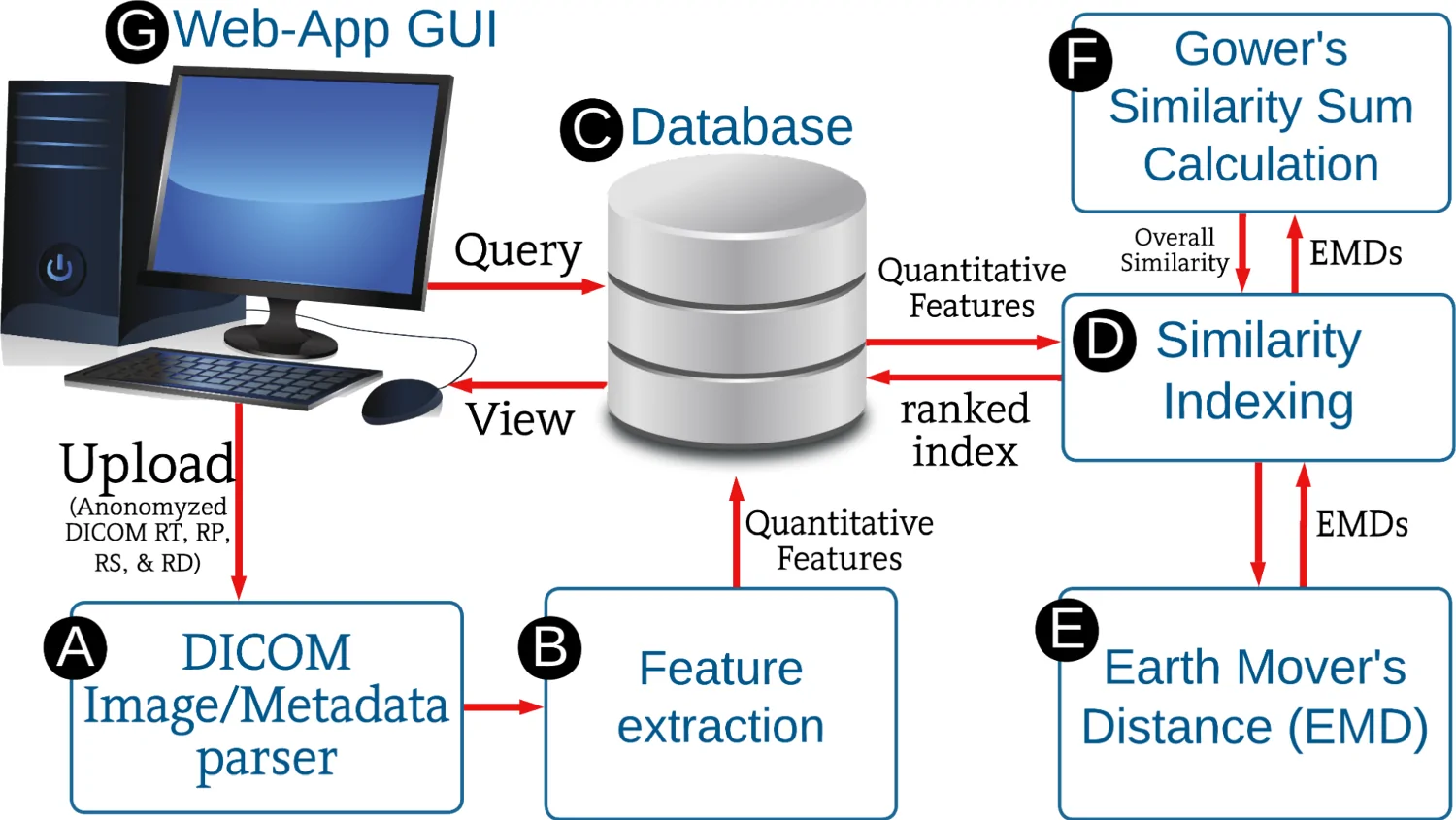
Radiotherapy treatment planning decision support system architecture

Our infrastructure’s architecture enables GTV-OAR constellation geometry-based similarity matching to identify retrospective best practice cases for reference during treatment plan creation. A database (Fig. 3c) stores and organizes the knowledge base of retrospective radiotherapy cases and associated metadata, including quantitative features and similarity measures. The data processing pipeline starts with a DICOM RT parser (Fig. 3a) to upload and organize data from retrospective and current cases. A feature extraction module (Fig. 3b) then calculates quantitative identifying features. For similarity indexing (Fig. 3d) our system calculates pairwise differences between corresponding quantitative features (Fig. 3e) and then computes an aggregate similarity sum from these metrics (Fig. 3f). The frontend web application (Fig. 3g) implements the clinical workflow intervention and decision support tool, allowing users to upload current cases and utilize identified similar retrospective cases.

### Data structure and database

Facilitating easy access through standardized structuring and storage of radiotherapy imaging and metadata is essential for clinical utility of our decision support infrastructure. To enable multi-institutional data use, the infrastructure is vendor neutral, incorporating and extending the DICOM standard. New radiotherapy techniques made available by the development of software defined radiation delivery systems in combination with informatics infrastructure improvements have facilitated use of previously collected clinical data. We continue this trend by expanding the informatics infrastructure to create, store, structure, and use target-OAR relational data for similarity matching. Our system architecture facilitates access to a database of retrospective best practice head and neck (H&N) cancer cases. This database also expands upon existing structures by storing and structuring the calculated similarity data.

**Fig. 4:**
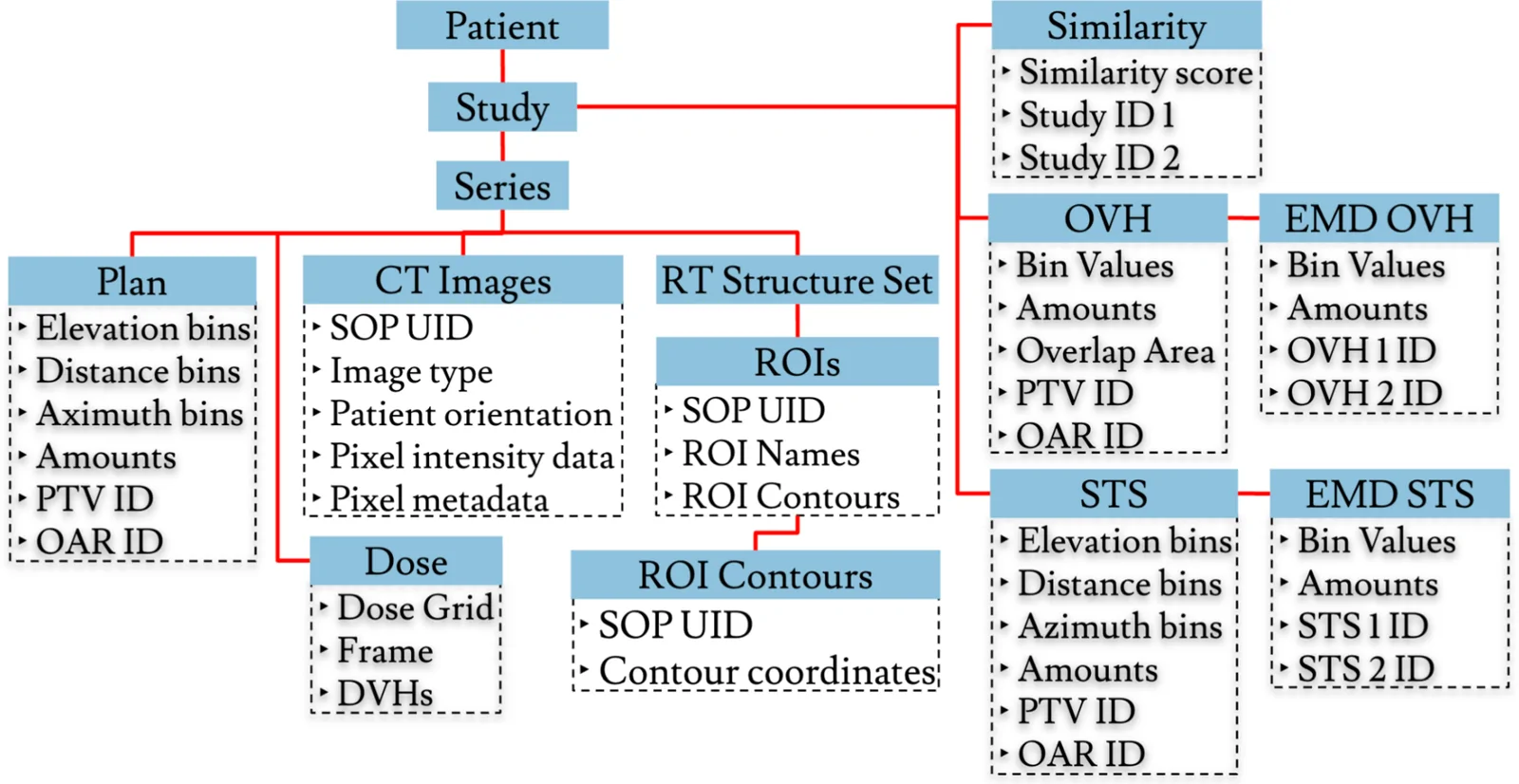
Radiotherapy treatment planning decision support database schema. The schema of the database stores and structures the DICOM RT data parsed from uploaded cases as well as the data created for anatomical structure characterization and similarity matching. Dose-value Histogram (DVH), Earth Mover’s Distance (EMD), Organ at Risk (OAR), Overlap Volume Histogram (OVH), Planning Target Volume (PTV), Region of Interest (ROI), Service–Object Pair Unique Identifier (SOP UID), and Spatial Target Signature (STS)

Our database schema (Fig. 4) processes and organizes two main data categories for similarity matching: DICOM RT and metadata. For DICOM RT data, our schema mirrors standard structures for DICOM CT images, RT dose, RT plan, and RT structure set. Organized access to 3D images and ROI models within the RT structure set allows geometry-based similarity matching methods. The metadata we generate during algorithmic similarity matching is structured and stored in relation to both the case and organ, enabling meaningful clinical use and accurate GTV-OAR constellation geometry matching.

### Multi-organ constellation data mining

Our system infrastructure enables matching radiotherapy cases based on GTV-OAR constellation similarity. This involves several steps supported by our full medical imaging informatic infrastructure, which provides structured storage and access to radiotherapy data (i.e., images and ROIs from DICOM RT structure sets) and the data we create from it (relational quantitative features, similarity metrics, and front end user credentialing). First, the GTV-OAR geometry is quantified using extracted features describing the GTV-OAR spatial relationship. For each case the quantitative features, an overlap volume histogram (OVH), and a spatial target signature (STS) are calculated between the tumor and each OAR. Second, corresponding features of the same type and OAR are paired between the two cases. Third, the similarity between matched quantitative features is calculated using an Earth Mover’s Distance (EMD). Finally, the overall similarity between two cases is computed as the Gower’s distance of all the EMDs between the two cases. Leveraging our newly developed infrastructure, we introduce a novel comprehensive geometric characterization of the GRV-OAR constellation using quantitative features designed to enhance similarity matching and treatment planning decision making.

#### Feature extraction

The GTV-OAR constellation geometry, which our system uses to determine similarity between cases, is described using two quantitative features calculated multiple times for each case. These features quantify the spatial and distributional relationship between the tumor volume (GTV) and one OAR. For each case, the system computes and stores the OVH and STS between the tumor and each relevant OAR defined in the DICOM RT structure set (e.g., for 15 OARs there will be 30 features).

The first feature is the OVH described by Kazhdan et al. [18]. This 1D histogram is constructed from the distribution of the distances from each voxel inside the OAR to the nearest point on the GTV surface. The OVH reflects geometric conditions that influence potential dose value histograms (DVH) in optimal plans, validating its efficacy by considerations clinicians use [18]. By using multiple OVHs from different OARs simultaneously, we are able to achieve a holistic measure of similarity.

**Fig. 5:**
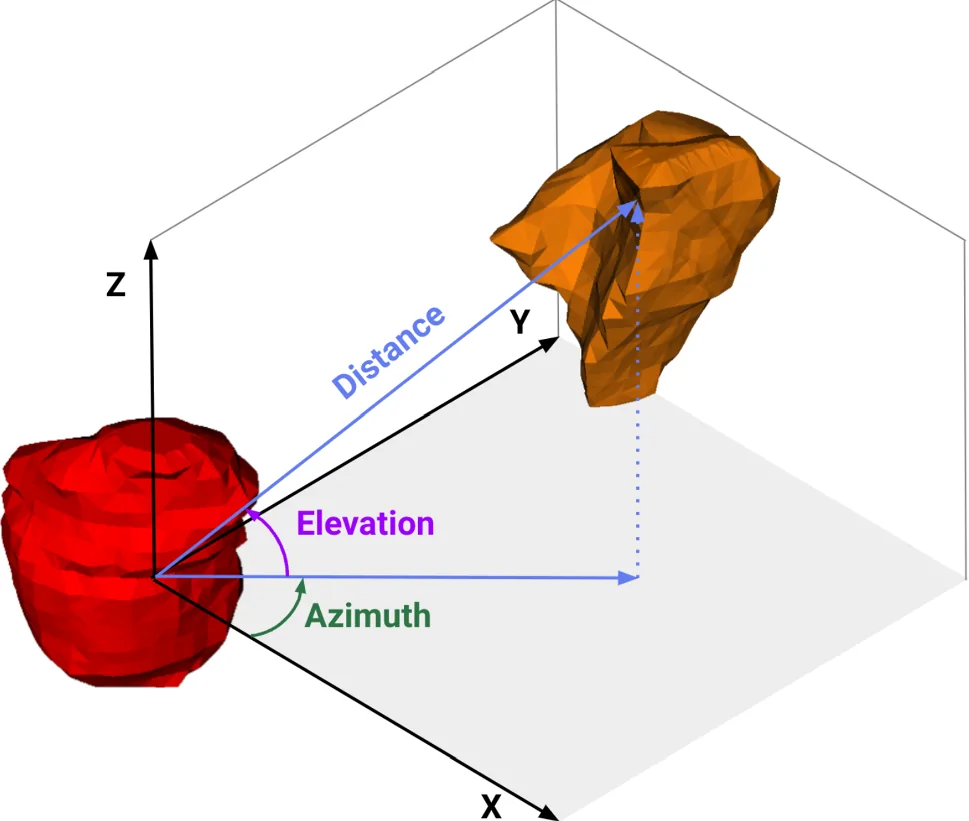
The spatial target signature (STS). A single vector from the OAR centroid pointing to a GTV voxel, with azimuth, elevation, and distance

The second feature is the spatial target signature (STS) described in Desphande et al. [17]. This 3D histogram represents the tumor volume’s spatial distribution relative to the OAR’s centroid (Fig. 5). Specifically, it is the histogram of the vectors (distance, azimuth, and elevation) pointing from the OAR centroid to each tumor voxel. The STS represents the tumor’s orientation, size, shape, and distribution relative to the OAR, aspects the OVH does not fully characterize. By utilizing both the STS and OVH for each OAR, we construct a more complete characterization of the GTV-OAR constellation.

#### Similarity matching

Our system infrastructure enables the calculation of overall similarity between two radiotherapy cases simultaneously using all relevant quantitative features. This comparison occurs in two steps. First we calculate the similarity between individual corresponding paired quantitative features (e.g., OVHs and STSs histograms for a specific OAR from two different cases) using the Earth Mover’s Distance (EMD). The EMD measures the similarity between two histograms by calculating the minimum “work” needed to transform one distribution into the other, solved as an optimized linear transportation problem [25]. This provides a more characteristic representation of the difference compared to simple absolute value differences and is a standard method for comparing histograms in radiotherapy.

Second, these EMD similarity scores are combined into a single similarity value using Gower’s Distance. Gower’s Distance calculates an overall similarity metric from different types of individual similarity measures, here the EMDs, by finding an evenly weighted sum of the normalized scores [26]. The weighting is proportional to the number of OAR the two cases have in common, normalizing the result and making calculated similarity equivalently comparable between case pairs with different numbers of OAR in common. By calculating a Gower’s Distance between a current planning case and every case in the knowledge, we generate a ranked similarity index. These similarity values form the index which allows our infrastructure to efficiently present to clinicians retrospective best practice cases with GTV-OAR constellation geometries similar to the current case.

### Decision support web application

To facilitate clinical use, we developed a frontend web application for our treatment planning decision support system. This web application was constructed using a Python Django framework connected to a MySQL database, with JavaScript and HTML shaping the user interface. Radiation oncologist and medical physicist clinical collaborators at University of Minnesota, and Cedars-Sinai Medical Center guided our web application design to ensure smooth integration into existing clinical workflows.

The system architecture is deployed within a containerized Docker environment hosted on secure USC IPILab servers behind USC’s Information Technology Services’ firewall to preserve data privacy and security. In case of specific institutional data requirements, the system is vendor neutral and designed to be easily deployed as a standalone application locally within any institution through containerization.

**Fig. 6:**
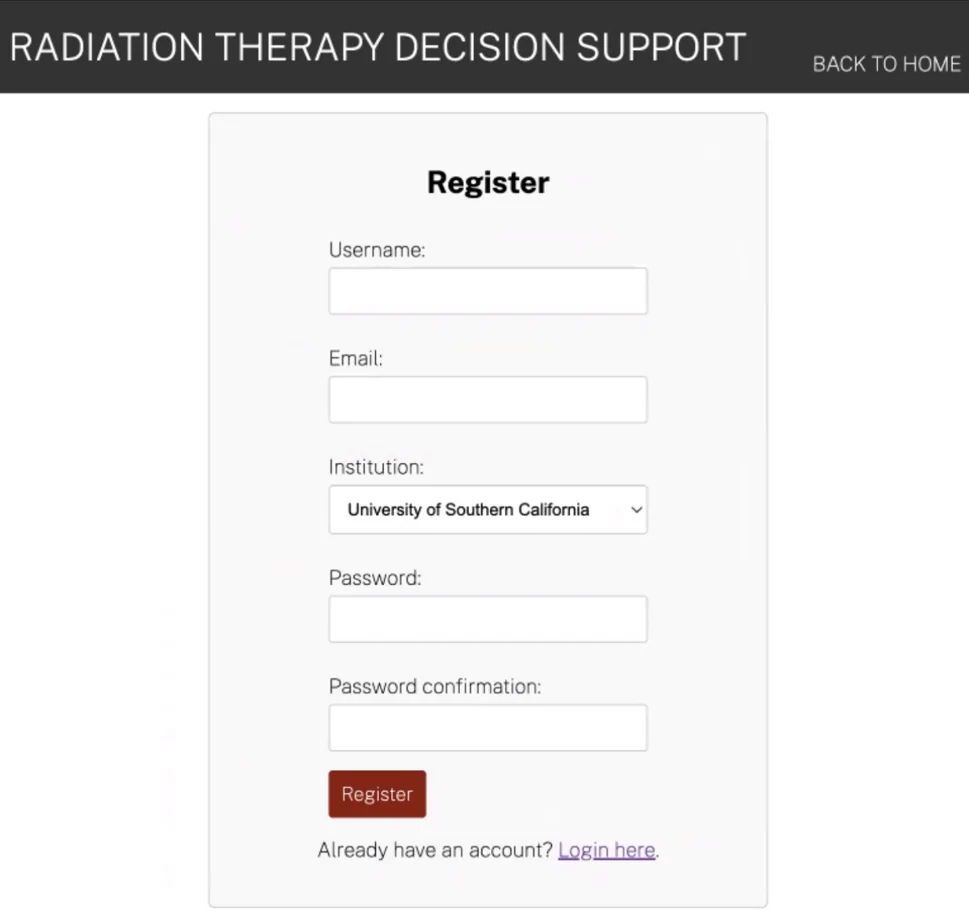
Web application login web page

Access to the application and its knowledge base is controlled through user credentialing and a “Login” page which enables multi-institutional deployment and use of this vendor neutral system (Fig. 6). Case and dataset access are regulated based on clinical user institution affiliation and specific authorizations. Highly restricted datasets can be limited to users from the originating institution or excluded from extra-institutional deployment.

The web application allows clinicians to upload planning cases to find retrospective best practice cases with similar GTV-OAR constellation geometries. To preserve privacy, only essential, non-identifying DICOM data (ROI contours, CT voxel intensity) required for similarity matching are parsed.

The web application also contains a “Patients” and a “Similarity” page. The “Patients” page tracks uploaded cases and the status of their asynchronous similarity calculation. The “Similarity” page interactively displays the most similar retrospective cases, and features side-by-side CT image sliders for visual comparisons. More details on the “Similarity” page as a decision support tool are discussed in the Clinical web application implementation section.

## Results

The GTV-OAR constellation geometry similarity matching-based radiotherapy treatment planning decision support system and infrastructure was evaluated for clinical use with the proposed workflow intervention and use with VMAT H&N RT cases. The project is a collaboration between the USC Imaging Processing and Informatics laboratory and clinicians at Cedars-Sinai Medical Center and University of Minnesota Departments of Radiation Oncology. The system was developed and tested using a knowledge base of pre-anonymized retrospective best practice cases, DICOM RT Plan (RP), RT Dose (RD), RT Structure Set (RS), and CT Image files, from collaborating institutions (Table 1). While initially developed with H&N volumetric arc therapy (VMAT) cases obtained under IRB EDR-103707, the infrastructure is designed to be able to provide treatment planning support for any cancer pathology, provided a sufficiently large knowledge base exists to provide insight for the specific GTV-OAR constellations that may present.

**Table 1:** Data sets of retrospective radiotherapy cases included in the system knowledge base

Data set	Number of cases	Type	Notes
Roswell Park CCC Buffalo 2017	97	VMAT H&N cancer (lower neck)	CT, RP, RD, RS and half contain PT studies
Roswell Park CCC Buffalo 2018	132	VMAT H&N cancer (lower neck)	CT, RP, RD, RS and all contain PT studies as well
Roswell Park CCC Buffalo 2019	35	VMAT H&N cancer (lower neck)	No RP files, some have RE and RI
Roswell Park CCC Buffalo 50	50	VMAT H&N cancer (lower neck)	CT, RP, RD, and RS
Roswell Park CCC Buffalo 18 5	5	VMAT H&N cancer (upper head)	CT, RP, RD, and RS
TUM	71	Tomotherapy H&N (lower neck)	CT and RS

**Fig. 7:**
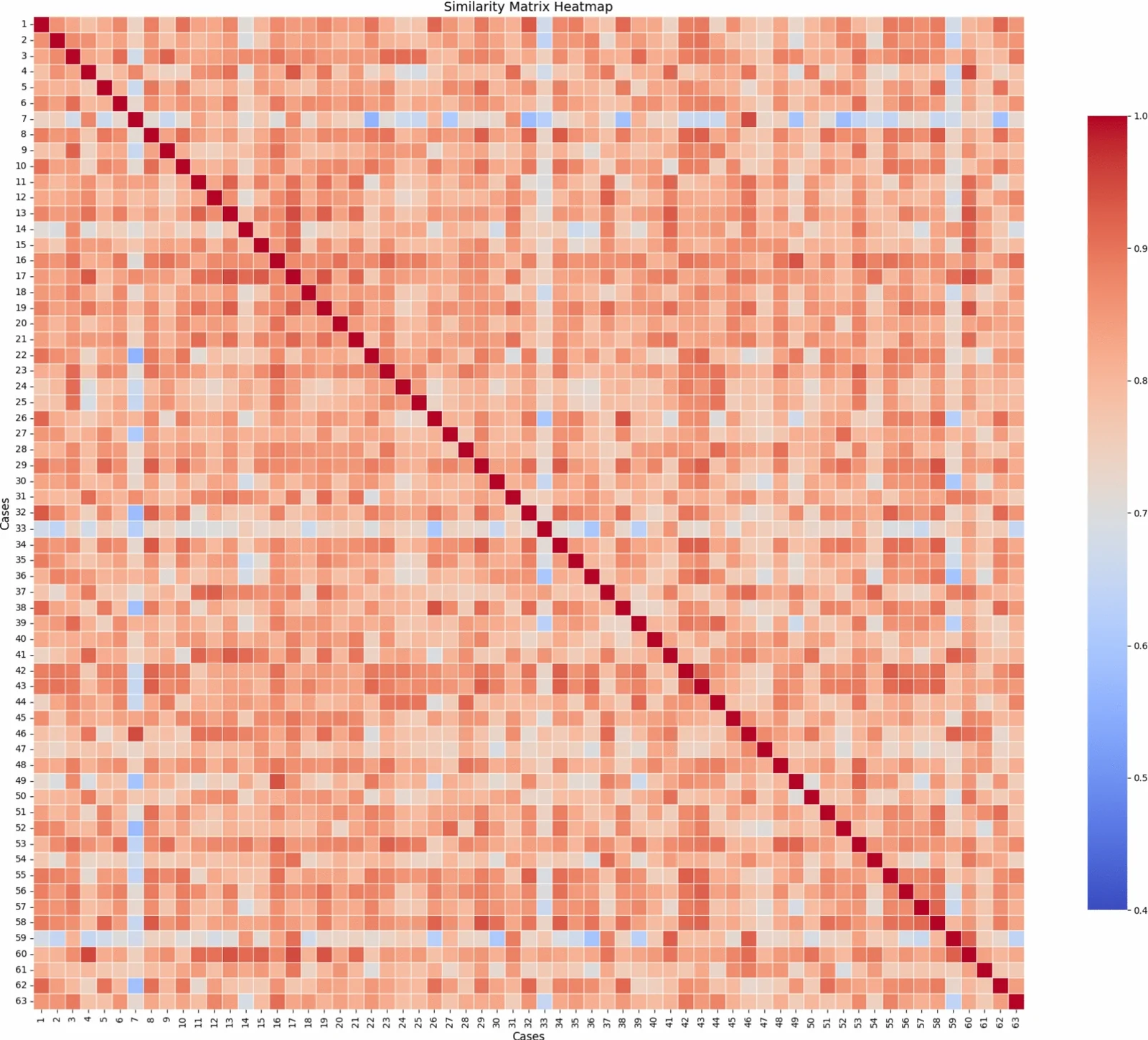
Knowledge base heat map of similarity between VMAT H&N cases. Sixty-three cases from the Buffalo 2017 dataset are shown in the heat map

We used the cases from these datasets to build the knowledge base and validate our system. While the DICOM format imposes standardization in case data, significant variation exists which required us to build a robust infrastructure capable of handling non-uniform data. Testing with cases from multiple institutions ensured the system could accommodate variations in uploaded planning cases and retrospective best practice case datasets. To validate the data pipeline and overall operation, all knowledge base cases were processed in the role of current planning cases (i.e., the similarity from each case was calculated to every other case in the database (Fig. 7)).

The resulting similarity relationships are visualized in a heatmap (Fig. 7). Analysis shows expected patterns: cases highly similar to a common third case were often similar to each other (e.g., cases 1, 26, 27 have high mutual similarity). This revealed clusters of cases with high mutual similarities, such as those centered around the similarities of case 13 and 17 or case 57 and 56, suggesting shared tumor position and structural anatomy. We developed a clustering method to further explore the grouping of cases to relate anatomical similarity to treatments and outcomes.

We further evaluated the infrastructure using clustering methods. K-means clustering, with silhouette analysis, served as an initial exploratory step, allowing investigation without rigid pre-specification of cluster numbers. Spectral clustering was subsequently used because it allowed better visualization and representation of discrete case clusters.

**Fig. 8:**
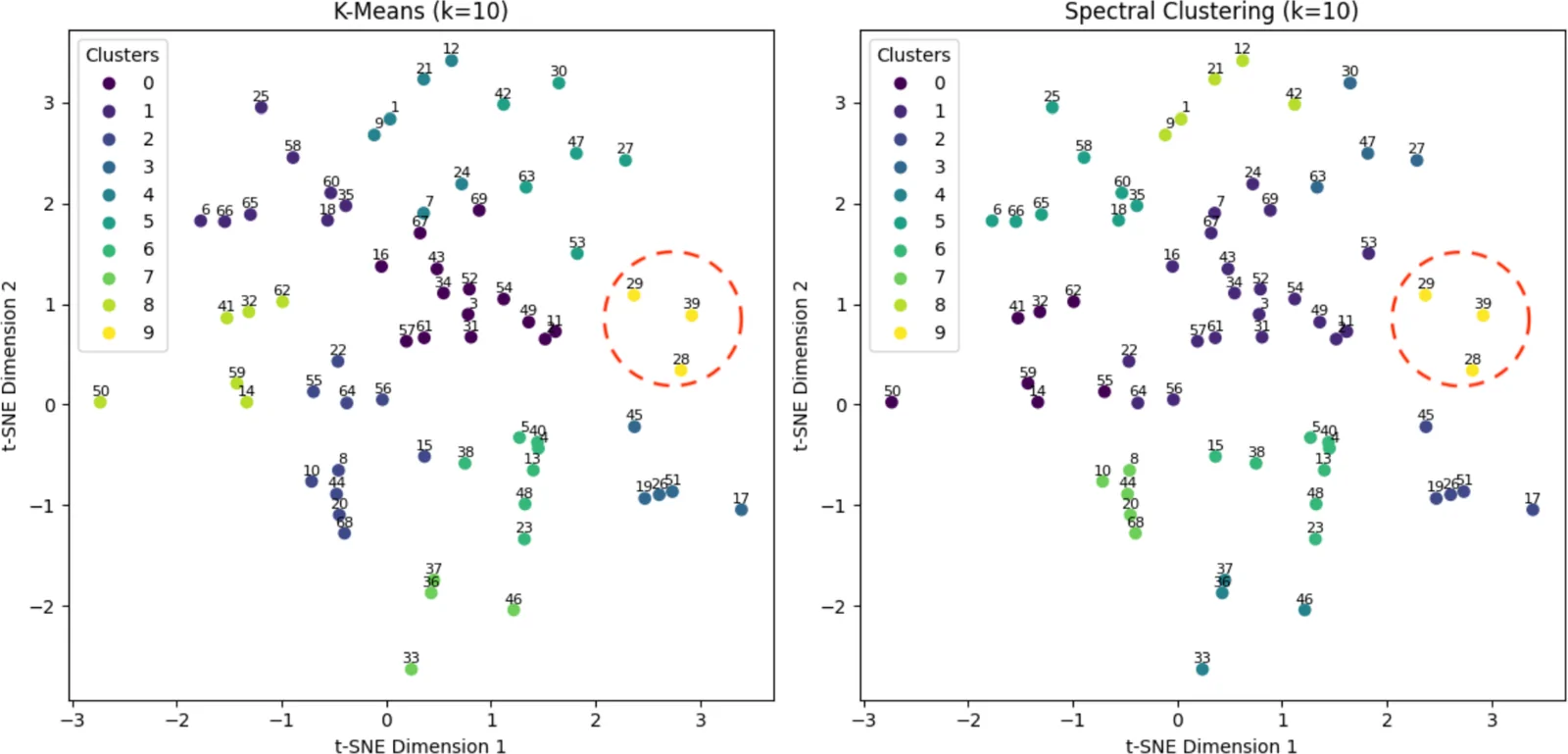
K-Means and Spectral clustering of CCC 2017 dataset H&N cases by GVT-OAR constellation similarity with t-SNE dimensionality reduction. One representative grouping, cluster 9, cases 29, 39, and 26, is demarcated with a red circle for emphasis

**Fig. 9:**
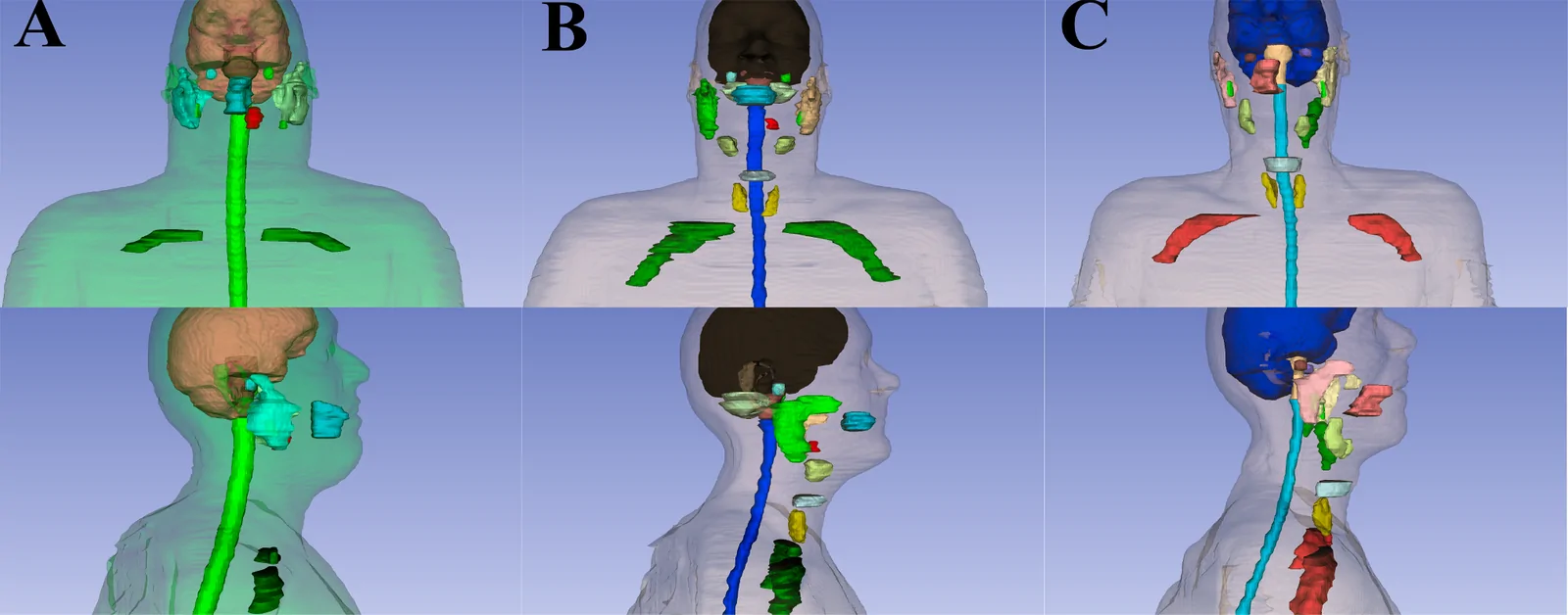
Three cases from cluster 9 identified through k-means and spectral clustering. Three cases respectively **a** 29, **b** 39, and **c** 28 with 3D frontal and sagittal views of the ROIs including OARs and tumor volume. The tumor volumes are left of midline at the level of the submandibular glands and respectively colored **a–b** red and **c** green. OARs in this visualization include the body, brain, spinal cord, parotid (left), parotid (right), tongue, cochlea (left), cochlea (right), thyroid, larynx, submandibular (left), submandibular (right), and brachial plexus

To graphically represent the clustering based on GTV-OAR constellation similarity, we used t-distributed stochastic neighbor embedding (t-SNE) dimensionality reduction to map the high-dimensional similarity data onto a 2D plane [27, 28]. Analysis of the Roswell Park CCC Buffalo 2017 H&N VMAT dataset identified ten distinct clusters (Fig. 8). Qualitative inspection confirmed similar tumor locations and GTV-OAR constellation characteristics within clusters. Investigation of one representative cluster, cluster 9 (Fig. 9), revealed three cases with similarly sized tumors (~ 10 mm high and ~ 12 mm in diameter) located consistently (~ 20 mm left of midline at the level of the submandibular glands), demonstrating high intra-cluster similarity.

This clustering analysis further validates the system’s successful operation and highlights next steps for development. Identifying groups with shared structural anatomy allows for associating treatment plans and configurations with outcomes within those groups. This could enable determining optimal plans for specific GTV-OAR geometries, benchmarking plan quality, and providing quality assurance, adding further utility to our decision support infrastructure. Continued research using this platform to cluster cases by geometric similarity will improve understanding of common tumor geometries, spatial relationships, and treatment correlations, demonstrating its function as both a clinical treatment planning decision support tool and a platform facilitating further research.

### Clinical web application implementation

Designed through consultation with clinical collaborators, our web application supplements standard treatment planning workflows, aiming to facilitate faster, easier creation of high quality plans. Our decision support infrastructure intervenes after ROI segmentation but before the initial treatment plan configuration phase.

**Fig. 10:**
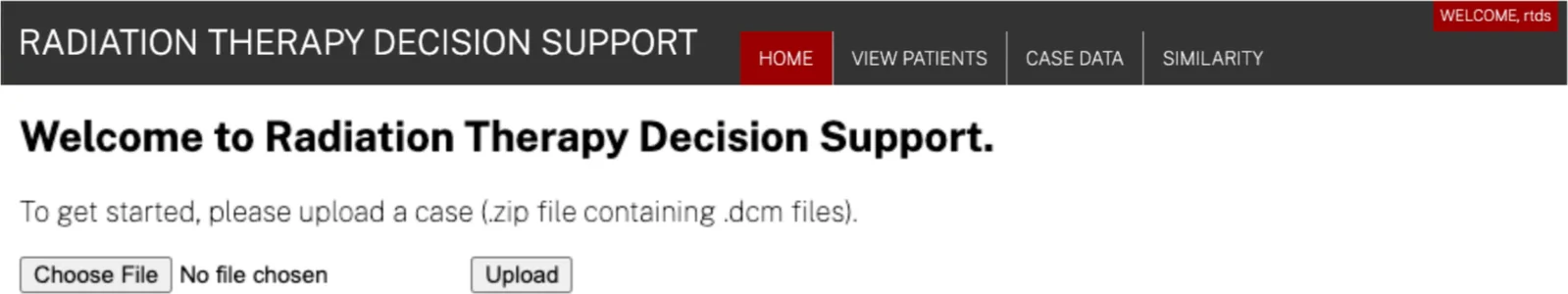
Web application upload web page

**Fig. 11:**
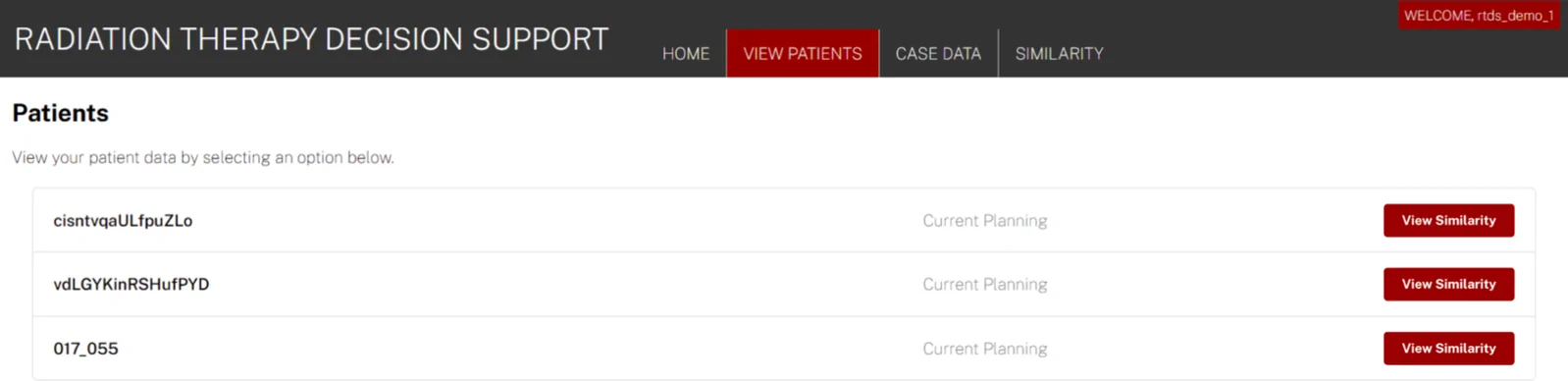
Web application patients web page

Instead of relying solely on clinical experience for the initial plan, clinicians upload the current planning case (zipped DICOM; RT Structure Set and CT images) to our web application (Fig. 10). The system automatically processes the case, extracting quantitative features and calculating the similarity index. The subsequent “Patients” page lists all current planning cases uploaded from the user’s institution (Fig. 11). Clicking the ‘View Similarity’ button for a specific case takes the clinician to the “Similarity” page to examine the identified similar retrospective cases.

**Fig. 12:**
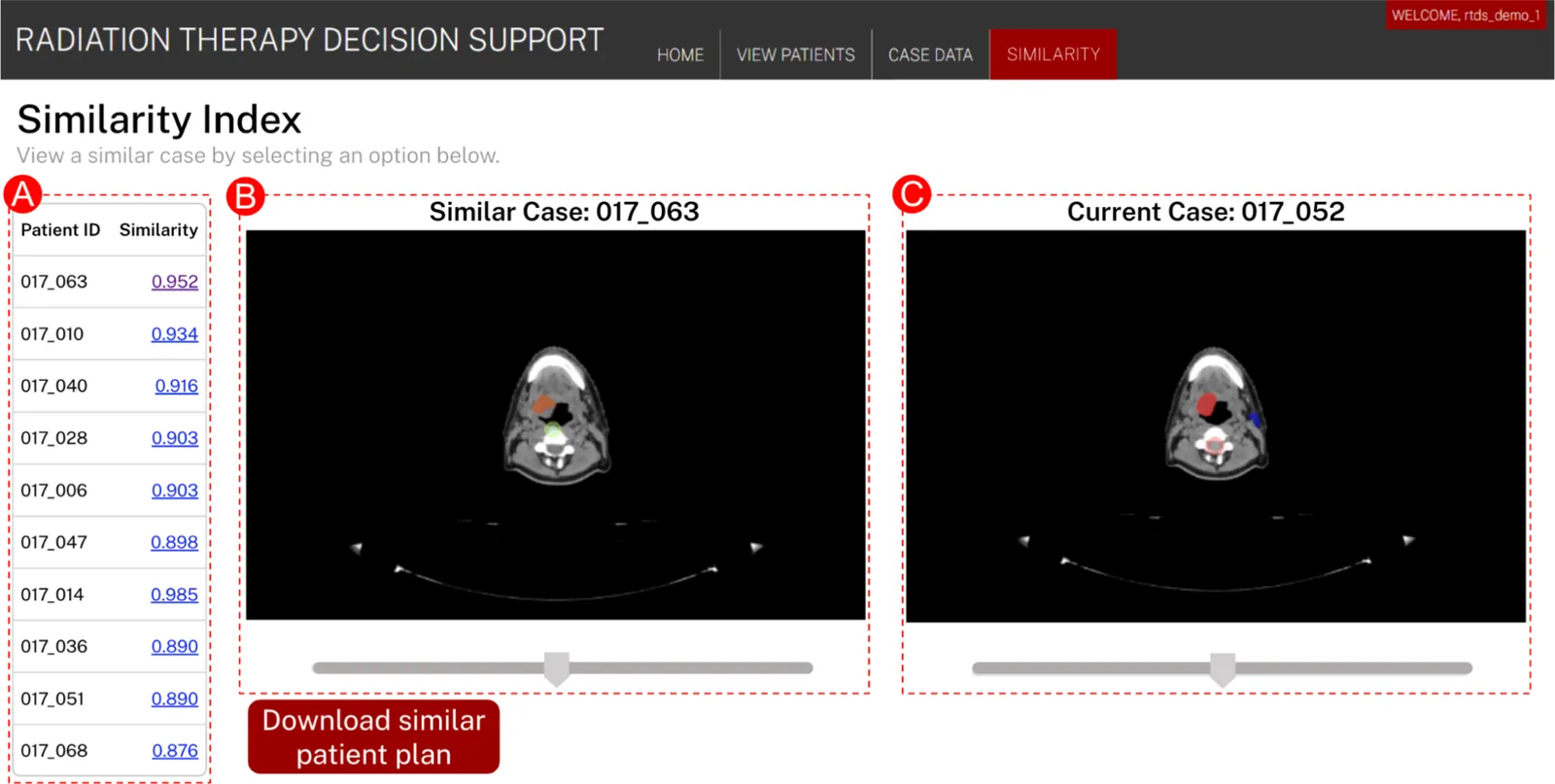
Similarity Index Viewing Page. Figure showing web application similarity viewing page.** a** Similarity index showing the ten most similar retrospective cases and their similarity. **b** CT Image slider of the currently selected similar retrospective case. **c** CT Image slider of the current planning case

Clinicians then use the “Similarity” page to view and select useful retrospective best practice knowledge base cases (Fig. 12). This page is the core element of our system decision support, allowing visual inspection of the identified similar cases in an interactive GUI. It features three main components: the similarity index (graphical list of cases ranked by similarity, (Fig. 12a)), a CT image slider for the current planning case (Fig. 12c), and a CT image slider showing a similar case selected from the similarity index (Fig. 12b). The sliders can be locked for synchronized scrolling, aiding comparison of OAR and tumor contours. The ROI contours for the tumor volumes and the OARs in common between the two visualized cases will be shown in the CT image sliders.

To use the “Similarity” page, clinicians peruse the list of similar cases (Fig. 12a), selecting cases for detailed examination. After selecting a case, its CT images appear in the similar case CT image slider (Fig. 12b). The clinician evaluates its similarity against the current planning case (Fig. 12c), exercising professional judgment considering the specific clinical requirements to determine usefulness as a reference. If unsuitable, this process can be repeated by selecting and comparing other cases. Once useful reference case(s) are identified, the clinician can download their DICOM files (CT image, RT Plan, RT Dose, and RT Structure set) with the download button. This similar case serves as a reference in the clinician’s preferred DICOM viewer or treatment planning software (TPS) during initial treatment plan creation, after which the standard clinical workflow resumes with the iterative optimization process.

While introducing additional steps, our system aims for minimal disruption to existing workflows. The time spent using our tool is expected to be offset by reduced initial configuration time and potentially fewer iterative optimization cycles. Future work includes analyzing clinical impact through quantitative similarity accuracy evaluation (phantom data, clinician rank similarity comparisons) and workflow studies (efficiency gains, usage patterns, and clinical usability and benefits feedback). We plan third-party plugin integration with popular TPSs, including Eclipse and Raystation, for a more seamless workflow intervention and reduced system use time cost.

## Conclusion

Our treatment planning decision support system matches radiotherapy cases based on GTV-OAR constellation geometry similarity, identifying retrospective cases for reference during initial treatment plan creation. The purpose of this system is to improve treatment plan quality, facilitate creation of high quality plans, enhance clinical workflows, and explore the relationships between tumor position, anatomy, and best practice treatments for different GTV-OAR constellation geometries. Use of this decision support system in the proposed workflow intervention will be clinically useful for the following reasons:

1. **Treatment plan standardization:** Presenting best practice treatments derived from structurally similar cases enables the system to increase standardization and optimization of initial beam field geometries by helping identify superior starting point beam configurations for specific tumor positions and OAR relationships.
2. **Edge/Outlier cases:** This tool offers utility for outlier cases where clinicians’ prior experience may be less applicable due to uncommon tumor geometry. Initial treatment plan creation relies on experience, making uncommon tumor positions and situations more difficult and time consuming. Acquiring knowledge for more rare outlier cases might take years or never happen. By using this system for uncommon GTV-OAR constellations, clinicians gain access to necessary knowledge, enabling faster creation of higher-quality plans for these new and difficult pathologies.
3. **Quality improvement and planning facilitation:** The system assists during initial beam creation, a phase in treatment planning neglected by current decision support tools. Poor initial plan configurations can prevent local optimization from identifying optimal plan configurations, limiting plan quality. Improving the quality of starting configurations can reduce required iterative optimization cycles and enhance achievable plan quality.
4. **Improved insight into proven superior treatment dosages and configurations:** Integrating outcome data (e.g., toxicity, survival) will enhance the system. It will identify not only treatment used for similar cases but also provide information on their efficacy. Combined with clustering analysis based on GTV-OAR constellation geometry similarity, this allows identification of superior treatment configurations and dosages for particular tumor locations, offering insight into treatment efficacy.

Our work builds on existing KBP and MIII practices by creating a DICOM standard-based infrastructure implementing holistic models of GTV-OAR constellation geometry matching. We extended existing data formats and structures to enable accurate and clinically usable geometric anatomical similarity matching. The system provides clinicians access to retrospective cases with similar GTV-OAR constellation geometry for use as references. Initially built using H&N VMAT data (due to complexity and OAR count), the infrastructure supports other external beam pathologies given a sufficient knowledge base. Additional datasets will continue to be acquired to power clustering quality assurance and benchmarking.

Future development will focus on refining and evaluating similarity matching algorithms. KBP models currently under development and evaluation with this infrastructure will incorporate quantitative features calculated from multiple different target dose, node, and tumor volumes to all OARs to better represent and identify similar cases by the spatial anatomy and the prescribed treatment. Machine learning KBP models are also being developed with target-OAR quantitative features and features representing the different node levels as input. The machine learning models are being trained by the dose similarity between cases which will enable the geometry-based model to differentially consider the different target-OAR relationships in a manner that reflects how they influence clinical determination of dose plans. More datasets of retrospective cases are being acquired through clinical collaborations to expand our multi-institutional knowledgebase. These datasets will focus on pathologies and modalities where there is a more beneficent use case for our system; where the beam number and angle selection is a harder, more complicated and important part of planning, such as in non-coplanar cases of SBRT or peripheral brain VMAT. Additional refinement and integration of the frontend web application with existing TPSs as third-party plug-ins will further improve system utility and usability.
